# Innate Immunity and the Role of Epithelial Barrier During *Aspergillus fumigatus* Infection

**DOI:** 10.2174/157339512800671985

**Published:** 2012-08

**Authors:** Elena Svirshchevskaya, Dmitrii Zubkov, Isabelle Mouyna, Nadia Berkova

**Affiliations:** 1 Shemyakin and Ovchinnikov Institute of Bioorganic Chemistry, RAS, Moscow, Russian Federation, Russia; 2 Aspergillus Unit, Pasteur Institut, Paris, France; 3 INRA; Agrocampus Ouest, UMR 1253, STLO, Rennes, France

**Keywords:** Aspergillosis, *Aspergillus fumigatus*, dectin-1, defensin, epithelial cells, fikolin-2, genetic factors.

## Abstract

Fungi are the most important eukaryotic infective agents in Europe which largely overpass parasite infections. Total number of people dying of fungal infection is increasing and this trend is likely to continue due to the increase in immunosuppressive treatments. The opportunistic pathogen *Aspergillus fumigatus* (Af) is a saprophytic filamentous fungus that can cause invasive pulmonary diseases in immuno-compromised hosts. In veterinary medicine aspergillosis is also a recurrent problem since it infects various species, birds are particularly susceptible. It propagates through airborne conidia (spores), which are inhaled into the small airways where they may germinate and initiate an infection. The host epithelium has permanent contact with the environment and a multitude of diverse microorganisms, resulting in a network of the host’s defense mechanisms. Pathogens use various strategies to invade epithelial barriers, to exploit eukaryotic host function to their own benefit and disseminate throughout the host using the epithelium as a reservoir. The current revue will discuss the ways how epithelial and innate immunity cells can contlol Af infection. We will focus on Af strategies for the host’s invasion, antifungal innate immune response and antimicrobial activities of the respiratory epithelial cells.

## INTRODUCTION

Host’s airways have a vast epithelial surface at the interface with the environment, therefore it is exposed to the multitude of the microorganisms. The respiratory epithelium plays an important role in the innate immune defense against various inhaled pathogens by sensing the signal from the external environment. Pathogenic microorganisms have evolved different mechanisms to survive in the host environment. The invasion of non-phagocytic epithelial cells; alteration of the host cell apoptosis; promotion of cell proliferation or conversely, inhibition of cell growth; and modulation of the cell differentiation by blocking the cell cycle progression are some of them. The mechanisms of the strategies employed by Af are not fully known. The latest investigations of the interactions between host epithelium and microorganisms suggest that the epithelium is not a simple mechanical barrier: epithelial cells recognize microorganisms and initiate appropriate signaling which contributes to the endocytosis of microorganisms and the attraction of innate immunity cells. It appears that capture of microorganisms by the epithelial cells is selective and that the different endocytic mechanisms may be enhanced by proinflammatory cytokines. The specificity of the recognition is illustrated by the various studies, showing that the epithelial cells distinguish the different morphotypes of the microorganisms [1]. Af is a saprotrophic fungus that grows on decaying vegetal and organic material and plays an essential role in recycling the earth carbon and nitrogen. Af produces uninucleated haploid conidia, several hundred of which are continuously inhaled daily during routine activities. Therefore, the respiratory tract is the obvious site of infection. There are several clinical pictures of aspergillosis, such as allergic bronchopulmonary aspergillosis, chronic necrotizing aspergillosis, and invasive aspergillosis. The manifestation of the different forms depends on the patient’s immune status and/or underlying lung diseases. In immunocompetent individuals, mucociliary clearance and phagocytic defence normally prevent the disease, while invasive pulmonary aspergillosis is a major concern in immunocompromised patients. In the respiratory tract, the epithelium provides various fast mechanisms as front-line defence in the protection of the airway and the lung parenchyma from colonization and infection. Cellular responses mediated by macrophages, neutrophils and dendritic cells, serve as the second line of innate defence against Af.

## MECHANISMS OF HOST INVASION

### Aspergillus Proteases

Af conidia retained in the respiratory tract during colonization, releases various metabolic components of the fungus to the lungs. It was shown that Af produces a variety of extracellular proteinase that are believed to be virulence factors in *Aspergillus*-related lung diseases [2-3]. Af proteinase have been shown to degrade human lung elastin at a higher rate than an equimolar amount of human neutrophil elastase. Human lung collagen, such as types I, II, and fibronectins were quickly digested by the Af serine proteinases [4]. Proteases in extracts of Af cause epithelial cell desquamation and release of proinflammatory cytokines [2, 3, 5]. It was shown that even at low concentrations Af fungal proteases present in which are crude extracts, induce morphologic changes, cell shrinking, cell desquamation, and production of IL-6 and IL-8 [3]. The effects of the proteases can be abrogated by protease inhibitors. Thus, proteases present in fungal extracts interact with epithelial cells, induce morphologic changes such as cell desquamation and activate proinflammatory cytokines production by epithelial cells. By causing cell detachment, fungal proteases reduce the effectiveness of the physical barrier function of the epithelium, thereby, facilitating the entry of antigens. The activated epithelium signals the mucosal inflammatory response against Af. In the absence of proteases recombinant Asp f 1, Asp f 2, Asp f 4, and Asp f 6 proteins were unable to induce strong IgE production, although they have been shown to be highly effective in the induction of antigen specific IgG [6].

### Modulation of Apoptosis

A major innate immune response to inhaled Af conidia is the synthesis of pro-inflammatory cytokines, including a proapoptotic tumor necrosis factor (TNF)-alpha. Modulation of host cell apoptosis has been reported to be one of the mechanisms whereby pathogens overcome host cell defenses. We have shown that Af conidia were able to inhibit staurosporin induced apoptosis in A549 pneumocyte II line, human tracheal epithelial 16HBE and primary human respiratory cells [7]. Inhibition of apoptosis by Af conidia was also observed when apoptosis was induced by co-cultivating A549 cells with activated human alveolar macrophages. Unlike Af conidia, conidia of *C. cladosporioides* as well as latex beads or killed Af conidia have no inhibitory effect on TNF-alpha or staurosporin-induced apoptosis. We have also studied the effects of *A. flavus*, *A. nidulans*, *A. niger* and *A. oryzae* conidia on human cells apoptosis [8]. Only *A. flavus* conidia but not of other species inhibited apoptosis of epithelial cells. These results suggested that suppression of apoptosis may play a role in reducing the efficacy of host defense mechanisms during infection with *Aspergillus* species.

## MECHANISMS OF HOST DEFENSE

### Humoral Innate Immunity

#### Surfactant Proteins in the Upper Layer of Mucus Epithelium

The upper layer of mucus epithelium is enriched with many different bactericidal and fungicidal factors called surfactants. Pulmonary surfactant consists of a lipid and protein complex essential for normal lung function. The proteins in surfactant complex belong to soluble pattern recognition receptors (PRRs) which participate in host defense by regulating pro-inflammatory cytokine production, chemotaxis, and tissue repair (Table **1**). In humans four surfactant proteins (SP) were identified: A, B, C and D. Both SP-A and SP-D are members of collectin family of proteins (**col**lagenous C-type **lectin**s), which have homologous amino-terminal collagen-like domains and multiple Ca^2+^-dependent carboxy-terminal carbohydrate recognition domains (CRDs) [9]. Several studies describe precise roles of surfactant proteins and collectins in antifungal response. Collectins bind pathogenic fungi *A. fumigatus*, *C. albicans *and others in calcium dependent manner [10, 11]. Their binding to *Aspergillus* conidia and hyphae was blocked by the excess of mannose, maltose, or 1,3-b-glucan [12, 13]. The studies carried out using SP-A^−/−^ or SP-D^−/−^ mice have revealed different roles of these collectins in surfactant homeostasis and pulmonary immunity.

#### SP-A

Compared to the WT mice, the SP-A^−/−^ mice have been found to have an increased susceptibility to a range of respiratory pathogens, including Group B *Streptococci*, *Staphylococcus aureus, Pseudomonas aeruginosa, Klebsiella* *pneumoniae*, respiratory syncytial virus, influenza A virus (IAV), *Mycoplasma pneumoniae*, *Pneumocystis carinii *and *Hemophilus influenzae* [14-18]. On the other hand, SP-A^−/−^ mice were nearly resistant to pulmonary hypersensitivity induced by Af allergens and even more resistant than WT mice to conidia challenge under corticosteroid induced immunosuppression [19-23].

#### SP-D

The SP-D^−/−^ mice show a delayed clearance of an exogenous challenge of pathogens, such as RSV and *P. carinii*, together with an exaggerated lung inflammation that can be restored by an exogenous administration of SP-D [24-27]. The SP-D^−/−^ mice were more susceptible than the WT ones to pulmonary hypersensitivity induced by Af allergens and the level of SP-D directly correlated with IgE level [20-23, 28]. The SP-D^−/−^ mice showed increased susceptibility to Af infection under immunosuppression [19-20]. Intranasal treatment with SP-D or rhSP-D was effective in ameliorating the pathology and mortality in the case of SP-D^−/−^ mice, whereas the SP-A treated Af-challenged SP-A^−/−^ mice showed increased mortality [28]. Taken collectively we can conclude that SP-A and SP-D play somehow opposite roles in *Aspergillus* infection and hyperreactivity, with SP-D being somehow more important for protection.

#### SP-B and SP-C

SP-B and SP-C are considered to be less important in lung resistance to pathogens. However, more recent data demonstrate that SP-B and C also take part in the innate defense. Genetic polymorphism in SP-B, C predisposes to severe lung infections induced by respiratory syncytial virus [29-30] while mice overexpressing SP-B had significantly decreased bacteria burden [31]. There only one publication by Haczku *et al. *[28] where SP-B and SP-C were analyzed during Af induced allergic response. It was shown that the expression of both SD-B and C was 50% decreased in mice. At the same time the level of SD-D was 9 times increased showing the opposite activity of SD-B, C and SP-D in allergic aspergillosis.

#### Blood Related Soluble Pattern Recognition Receptors

Upon inhalation Af conidia first encounter upper layer of mucus epithelium where they are entrapped by surfactant system. Those conidia which bypass this barrier are further collected by PRRs circulating in blood such as mannose binding lectin, ficalins, and pentraxins.

#### MBL

The main blood related collectin is mannose binding lectin (MBL) which also is present in the lungs. Binding by MBL of Af is inhibited by the excess of mannose (Table **1**). MBL was shown to increase survival in the model of invasive aspergillosis [32]. MBL deficiency is a risk factor for invasive aspergillosis in immunocompromised patients [33-34]. However, in immunocompetent mice knock-out of MBL gene led to an increased survival of mice, the fact which was explained by indirect attenuation of neutrophil reaction to Af challenge [35].

#### Ficolins 

Recently a new family of soluble PRRs, called ficolins, was discovered [36]. Three types of ficolins were identified in humans: ficolins 1-3 [36-38]. All ficolins are expressed at high level in liver and lungs. Their concentration in blood vary from 60 ng/ml (for ficolin-1) to 5 (for ficolin-2) and 25 (for ficolin-3) μg/ml. Among ficolins only ficolin-2 takes part in the recognition of Af [37] due to the binding to 1,3-β-d-glucan expressed by Af.

#### Long Pentraxin 3 (PTX3)

PTX3 is a soluble pattern recognition molecule playing a non-redundant role in resistance against Af [39]. A detailed molecular analysis has shown that PTX3 N-terminal domain was responsible for conidia recognition however the full-length molecule was necessary for opsonization. The protective activity of PTX3 against Af was dependent on FcγR-mediated recognition. PTX3 is present in blood in low quantities however it is quickly secreted by activated neutrophils [40]. PTX3 is shown to bind Ficolin-2 and enhance Ficolin-2-dependent complement deposition on the surface of Af [41]. It is likely that PTX3 and Ficolin-2 may recruit each other when they are immobilized on pathogens.

#### Secretory Mucosal IgA

Besides surfactants, dimeric IgA represents one of the most important components of innate system in mucosal secretions. However many patients with IgA deficiency are asymptomatic. Sinopulmonary infections of the respiratory system are the most common findings in individuals with IgA deficiency [42]. These infections are mostly due to bacteria, e.g., *H. influenzae* and *S. pneumoniae*. It is unlikely that mucosal IgA plays a role in anti-fungal response.

#### Cellular Innate Immunity

Type I collectins often form multimers which bind a wide range of pathogens easier than monomers. Collectins enlarge pathogens for phagocytes, and even damage bacterial membranes [43, 44]. As a result of pathogen opsonization with collectins alveolar macrophage engulf pathogens quicker, produce monokines at higher level and stimulate chemotaxis of neutrophils [45].

#### Resident Alveolar Macrophages (RAMs)

**Fig. (1):**
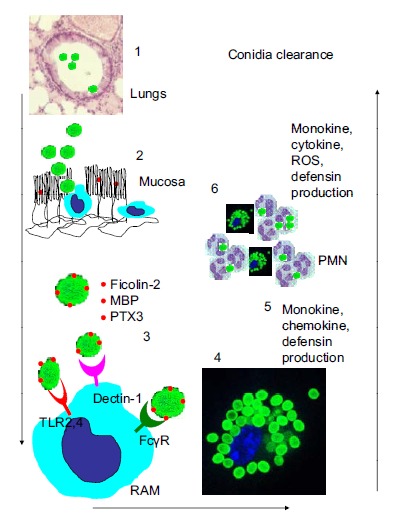
Upon inhalation resting conidia penetrate the alveoli (1) and are entrapped by mucous where (2) they are opsonized by Fikolin-2 which binds directly to 1,3-β-d-glucan, and to a lesser extent by MBP bound to galactomannose. Fikolin-2 bound to Af conidia is fixed additionally by PTX3. This complex is mostly washed out by ciliaric movement into the gut. (3) Some conidia are also grabbed by RAMs residing between ciliaric cells. RAM cells express FcγR able to bind Fikolin-2-PTX3 complex. Besides FcγR, RAMs also express Dectin-1 *via* direct interaction with surface exposed 1,3-β-d-glucan. This strong interaction induces phagocytosis of Af conidia (4) leading to intracellular digestion. When RAMs face high antigenic load whey start production of toxic molecules (5) like ROS and defensins simultaneously signaling by chemokines and monokines to central innate immune system for help. The major role in case of severe Af infection belongs to PMN (6) which migrate to the infection site and quickly phagocyte Af conidia which at that time germinate.

Among phagocytes resident alveolar macrophages (RAMs) represent the front line of armored defense in normal conditions. They phagocyte, ingest and kill inhaled Af conidia without activation and signaling [46, 47]. RAMs differ from blood derived or resident peritoneal macrophages by phenotype [48]. RAMs express β-glucan receptor dectin-1 at the highest level, while CD11b and F4/80 markers are almost absent on their surface. The fungicidal mechanism of RAMs against collectin-opsonized conidia is likely due to the oxidative burst generated during phagocytosis. They can control massive infection only for a short time [49]. However when their digestion capacity is overloaded during massive infection they initiate production of IL-1, TNF-α, and MIP1a in high amounts which stimulate neutrophil recruitment and subsequent antigen-specific immunity [50, 51] (Fig. **1**).

RAMs specifically recognize pathogens including fungi by cell associated PRRs. RAMs express toll-like receptors (TLR), nucleotide oligomerization domain (NOD)-like receptors, and C-type lectin receptors (CLR).

#### TLRs

In pulmonary aspergillosis, TLR2 and TLR4 have no role in immunocompetent animals, although TLR2^−/−^and TLR4^−/−^ are susceptible to invasive aspergillosis induced in immunosuppressed mice [52-54]. RAMs express TLR2 (Table **2**), which recognizes specific fungal cell wall motifs displayed during the conidial and hyphal stages [55]. The second TLR, important for antifungal response, is TLR4 expressed at the highest level by polymorphonuclear leukocytes (PMN). PMN also express TLR2 (Table **2**). Furthermore, Netea *et al*. [55] demonstrated that macrophages responded differently to conidia and hyphae. It appears that whereas TLR2 recognizes both conidia and hyphae, TLR4 only detects conidia. Upon ligation, TLRs initiate a signaling cascade *via *binding MyD88, consequent activation of transcription factor NF-κB and the production of cytokines and chemokines that prime innate and adaptive immunity [56]. The signaling pathway is analogous to the one triggered by IL-1β and intracellular domain of most TLRs is the same as of IL-1β receptor. Upon stimulation, MyD88 recruits IRAK-4 to TLRs through interaction of the death domains of both molecules, and facilitates IRAK-4-mediated phosphorylation of IRAK-1.

#### NOD-Like Receptors

NOD-like receptors are located in cytoplasm and bind microorganisms after they invade the host cell cytoplasm. There are no data that NOD-like receptors are involved in antifungal response in respiratory tract.

#### C-Type Lectin Receptors (CLR)

CLRs recognize pathogen associated molecular patterns composed of carbohydrate residues. Collectins described above belong to soluble CLR. C-type lectin receptors are a large superfamily of transmembrane polypeptides with multiple CRDs. This group includes the mannose receptor, phospholipase A2 receptor, DEC-205 and others.

#### C-Type Lectin-Like (CTLL) Receptors

CTLL receptors exhibit high similarity to the CRDs or exhibit the C-type lectin motif however most of them either do not bind carbohydrates, or bind them in a noncation-dependent manner. This group includes dendritic cell-specific ICAM-3-grabbing nonintegrin (DC-SIGN), langerin, macrophage galactose-type C-type lectin, and the phagocyte-associated C-type lectins 1 and 2 (dectin-1 and dectin-2), among others. CTLL receptors of all groups are thought to be involved in the recognition of pathogens and subsequent generation of the innate immune defense. It looks like dectin -1 and 2 are the most important for antifungal response. Dectin-1 recognizes β-glucan which is expressed in swollen but not resting conidia [12-13].

#### Dectin-1

Several reports demonstrate that expression of dectin-1 by RAMs and PMN is strongly required for the initiation of immune response [13, 57-58]. It is likely that dectin-1 participates in early killing of swollen conidia rather than hyphae. In pulmonary aspergillosis germinating Af conidia in the lungs express β-glucan, and dectin-1 mediates cellular infiltration and fungal killing [59]. Dectin-1^−/−^ mice have lower cytokine and chemokine production in the lungs after intratracheal infection, resulting in impaired neutrophil recruitment and increased susceptibility to Af [60]. Recently Leal *et al*. [61] developed a murine model in which red fluorescent protein (RFP)-expressing Af conidia were injected into the corneal stroma, and aspergillus induced keratitis progression as well as fungal survival were tracked over time. It was shown that dectin-1, TLR4, MyD88, and IL-1R1 were critical components of protective immune response while MD-2, TLR2, TIRAP or TRIF demonstrated no role.

#### Dectin-2

The role of dectin-2 in the response to Af is not well studied. However recently it was shown that Af extract stimulated a rapid production of proinflammatory lipid mediators cysteinyl leukotrienes (Cys-LT) from mouse bone marrow-derived dendritic cells [62]. Cys-LT production in BMDCs from wild-type mice was abolished in BMDCs from FcRgamma-/- mice, implicating either Dectin-2 or DC immunoactivating receptor. Transfection of each receptor in bone marrow-derived mast cells revealed that only Dectin-2 mediated cys-LT production stimulated by Af. Only lung CD11c+ cells, but not peritoneal or alveolar macrophages, generated cys-LTs in response to Af. These findings place Dectin-2 among the C-type lectin receptors that activate arachidonic acid metabolism and identify the Dectin-2/FcRgamma/cys-LT axis as a mechanism by which Af may activate innate immune cells to promote allergic inflammation.

#### Polymorphonuclear Neutrophils (PMN)

The second line of lung defense is mediated by neutrophils which start lung infiltration quickly [63]. Neutrophils express TLR 1, 2, 4 and 6 at high density. Among them only TLR2 and TLR4 are important for the recognition of Af [64-67]. TLR4 was shown to recognize either conidia or swollen conidia, but not hyphae [64-65]. It has therefore been proposed that the loss of TLR4-mediated proinflammatory signals during germination from conidia to hyphae represents an escape mechanism of Af from the host defense.

PMN secret many bioactive compounds upon stimulation *via *TLR system including PTX3, one of nonredundant mediators of immune response to Af. PTX3 is stored in specific granules and undergoes release in response to microbial recognition and inflammatory signals. Released PTX3 can partially localize in neutrophil extracellular traps formed by extruded DNA. Eosinophils and basophils do not contain preformed PTX3. PTX3-deficient neutrophils have defective microbial recognition and phagocytosis, and PTX3 is nonredundant for neutrophil-mediated resistance against Af. Thus, neutrophils serve as a reservoir, ready for rapid release, of the long PTX3, a key component of humoral innate immunity with opsonic activity. The critical role of neutrophils has been substantiated by the high susceptibility to Af infection of patients with severe and prolonged neutropenia.

####  Epithelial Cells

Host epithelium exposed to the microorganisms signals the presence of infection. Epithelial cells are not armored to digest pathogens however a growing body of evidence suggests that the epithelial cells play an important role in inflammatory process by secreting effector molecules such as surfactant proteins, cytokines or antimicrobial peptides [68].

#### Cytokines

The pathophysiologic mechanisms are mediated by a direct interaction of Af with airway epithelial cells, followed by an immunologic inflammatory response to pathogen. Epithelial cells are the first to encounter pathogens and express various cytokines. They produce the earliest wave of cytokines, which in turn trigger local inflammatory and systemic responses [69]. The first cytokines made in this cascade are interferon α (IFN-α), tumor necrosis factor α (TNFα), interleukin (IL) 1α, and IL-1β, soon followed by IL-6 and a variety of chemotactic cytokines such as IL-8 (KC in mouse), a neutrophil attractant, monocyte chemoattractant proteins (MCPs), and macrophage inflammatory proteins (MIPs) [70].

#### β-Defensins

β-Defensins are likely to be the major soluble effector molecules produced by epithelial cells. The defensin family of antimicrobial peptides is an evolutionary conserved group of small cationic peptides involved in the innate immune system of plants and animals. They are divided into α-, β- and θ-defensins, which differ from one another by the spacing and connectivity of their six cystein residues [71]. Human β-defensins (hBD) are characteristic of epithelial tissue [72-75]. Some of these defensins are tissue-specific, whereas others are expressed in the epithelium of different origins: hBD1 is expressed in most epithelial cells [76, 77], while hBD2 is most commonly expressed in the lung and thymus [78, 79]. Newly discovered defensin hBD9 was found to be ubiquitously expressed in most tissues. Inducible hBD2 expression by the epithelial cells exposed to microbial pathogens is well documented [80]. Direct killing of microorganisms by human defensins was described [73-75]. Killing of Af by rabbit neutrophil cationic peptides [81], as well as antifungal activities of hBD2 against Af [82], have been reported in *in vitro *experiments. Moreover, the expression of human drosomycin-like defensins, which display a broad spectrum of activity against Af, was found in several human tissues [83].

Recently using RT-PCR and real time PCR we showed the expression of hBD2 and 9 genes in bronchial epithelial 16HBE cells and A549 pneumocytes exposed to Af [1]. The expression was higher in cells exposed to swollen conidia compared to resting conidia or hyphae. The presence of hBD2 was also revealed using immunofluorescence. Cells labelling with anti-hBD-2 antibody showed a positive immunofluorescence signal around engulfed conidia but not around hyphae suggesting co-localisation of hBD2 and digested conidia. These findings provide evidence that respiratory epithelium might play an important role in the immune response during Af infection.

Additionally to the direct microbicidal activity [73, 75, 80, 84] microbial peptides have other activities such as the chemoattraction of immature dendritic cells, T cells and monocytes, as well as activation of the professional antigen-presenting cells [85], which might contribute to the regulation of host adaptive immunity against microbial invasion.

## CONCLUSIONS

Collecting the data on the role which different soluble or cell associated molecules play in Af clearance and infection resistance we have selected the most important among many which are summarized in Table **3**. The sequence of events involved in Af conidia traffic in bronchi is also depicted in Fig. (**1**). Upon inhalation resting conidia penetrate the alveoli and are entrapped by mucous where they are opsonized by Fikolin-2 which binds directly to 1,3-β-d-glucan, and to a lesser extent by MBP bounding to galactomannose. Fikolin-2 bound to Af conidia is fixed additionally by PTX3. Complexed Af conidia are mostly washed out by ciliaric movement into the gut. Some conidia are also grabbed by RAMs residing between ciliaric cells. RAM cells express FcγR able to bind Fikolin-2-PTX3 complex. Besides FcγR, RAMs also express Dectin-1 which interacts directly with 1,3-β-d-glucan exposed on the surface of conidia. The final and less important player is TLR4 which mostly binds peptidoglycans. This strong interaction induces phagocytosis of Af conidia leading to their intracellular digestion. When RAMs face high antigenic load whey start production of toxic molecules like ROS and defensins and simultaneously signal by chemokines and monokines to central innate immune system for help. The major role in case of severe Af infection belongs to PMN which migrate to the infection site and quickly phagocyte Af conidia which at that time begin to germinate. 

## Figures and Tables

**Table 1.** Specificity of Collectins

C-Type Lectins	Binding Moiety	*Aspergillus fumigatus PPRs*	Reference
Surfactant protein A	acetyl mannosamine, L-fucose, maltose, lipids	Mannose, maltose 1,3-b-glucan	[43]
Surfactant protein D	D maltose, mannose, glucose	Maltose, mannose	[43]
Ficolin-1 (M)	GlcNAc, GalNAc, sialic acid	-	[38]
Ficolin-2 (L)	1,3-β-d-glucan; *N*-acetylmannosamine, GlcNAc, GalNAc	1,3-β-d-glucan	[37, 86]
Ficolin-3 (H)	GlcNAc, GalNAc, D-fucose	-	[86]
Mannose binding lectin	N-acetyl glucosamine; L-fucose, mannose, N-acetyl mannosamine	Mannose	[43]
Long pentraxin 3	Fikolin-2	indirect	[39, 41]

**Table 2.** Distribution of Various TLRs on Different Immune Cells

Type of PRR*	PMN**	Macrophages	Dendritic Cells	Citation
TLR1	**++++**	+	+	[87, 88]
**TLR2**	++++	++++	+	[88, 89]
TLR3	-	+	**++++**	[87, 88]
**TLR4**	++++	++	+	[87, 88]
TLR5	-	+	**++++**	[87, 88]
TLR6	**++++**	+	+	[87]
TLR7	-	**++++**	+	[87]
TKR8	-	+	**++++**	[87]
TLR9	-	-	-	[87]
TLR10	-	-	-	[87, 89]

* PRR – pattern recognition receptor.

** PMN – polymorphonuclear leukocytes.

**Table 3.** Molecule Patterns Associated with AF Clearance

	Binding	Target	Citation
**Humoral Factors**			
SP-A, B, C, D	-	-	[20, 28]
MBL	±	galactomannan	[43]
Ficolin-1,3	-		
Ficolin-2	±	1,3-β-d-glucan	[37, 86]
PTX3	++		[39, 41]
IgA	-		
**Cell Associated Factors**			
Dectin-1	++++	1,3-β-d-glucan	[13, 59, 60]
Dectin-2	-		
Dectin-3	-		
TLR2	+	1,3-β-d-glucan	[88, 89]
TLR4	++	1,3-β-d-glucan	[87, 88]
